# Nested patterns of commensals and endosymbionts in microbial communities of mosquito vectors

**DOI:** 10.1186/s12866-024-03593-x

**Published:** 2024-10-26

**Authors:** Justė Aželytė, Apolline Maitre, Lianet Abuin-Denis, Alejandra Wu-Chuang, Rita Žiegytė, Lourdes Mateos-Hernandez, Dasiel Obregon, Vaidas Palinauskas, Alejandro Cabezas-Cruz

**Affiliations:** 1 Nature Research Centre, Akademijos 2, Vilnius, LT-08412 Lithuania; 2 Laboratoire de Santé Animale, ANSES, INRAE, Ecole Nationale Vétérinaire d’Alfort, UMR BIPAR, Maisons-Alfort, F-94700 France; 3 Laboratoire de Recherches Sur Le Développement de L’Elevage (SELMET-LRDE), INRAE, UR 0045, Corte, 20250 France; 4 Laboratoire de Virologie, Université de Corse, EA 7310, Corte, France; 5 Animal Biotechnology Department, Center for Genetic Engineering and Biotechnology, Avenue 31 between 158 and 190, P.O. Box 6162, Havana, 10600 Cuba; 6 School of Environmental Sciences, University of Guelph, Guelph, ON N1G 2W1 Canada

**Keywords:** Mosquitoes, *Escherichia-Shigella*, *Wolbachia*, Nestedness theory, Community assembly

## Abstract

**Background:**

Mosquitoes serve as vectors for numerous pathogens, posing significant health risks to humans and animals. Understanding the complex interactions within mosquito microbiota is crucial for deciphering vector-pathogen dynamics and developing effective disease management strategies. Here, we investigated the nested patterns of *Wolbachia* endosymbionts and *Escherichia-Shigella* within the microbiota of laboratory-reared *Culex pipiens* f. *molestus* and *Culex quinquefasciatus* mosquitoes. We hypothesized that *Wolbachia* would exhibit a structured pattern reflective of its co-evolved relationship with both mosquito species, while *Escherichia-Shigella* would display a more dynamic pattern influenced by environmental factors.

**Results:**

Our analysis revealed different microbial compositions between the two mosquito species, although some microorganisms were common to both. Network analysis revealed distinct community structures and interaction patterns for these bacteria in the microbiota of each mosquito species. *Escherichia-Shigella* appeared prominently within major network modules in both mosquito species, particularly in module P4 of *Cx. pipiens* f. *molestus*, interacting with 93 nodes, and in module Q3 of *Cx. quinquefasciatus*, interacting with 161 nodes, sharing 55 nodes across both species. On the other hand, *Wolbachia* appeared in disparate modules: module P3 in *Cx. pipiens* f. *molestus* and a distinct module with a single additional taxon in *Cx. quinquefasciatus*, showing species-specific interactions and no shared taxa. Through computer simulations, we evaluated how the removal of *Wolbachia* or *Escherichia-Shigella* affects network robustness. In *Cx. pipiens* f. *molestus*, removal of *Wolbachia* led to a decrease in network connectivity, while *Escherichia-Shigella* removal had a minimal impact. Conversely, in *Cx. quinquefasciatus*, removal of *Escherichia-Shigella* resulted in decreased network stability, whereas *Wolbachia* removal had minimal effect.

**Conclusions:**

Contrary to our hypothesis, the findings indicate that *Wolbachia* displays a more dynamic pattern of associations within the microbiota of *Culex pipiens* f. *molestus* and *Culex quinquefasciatus* mosquitoes, than *Escherichia*-*Shigella*. The differential effects on network robustness upon *Wolbachia* or *Escherichia-Shigella* removal suggest that these bacteria play distinct roles in maintaining community stability within the microbiota of the two mosquito species.

**Supplementary Information:**

The online version contains supplementary material available at 10.1186/s12866-024-03593-x.

## Background

Mosquitoes, as obligate hematophagous insects, are significant vectors for various pathogens, posing substantial health threats to humans and animals alike [1]. Among mosquito species, *Culex pipiens* f. *molestus* and *Culex quinquefasciatus* are of particular importance due to their role in transmitting diseases such as avian malaria, West Nile virus [2] and filariasis [3], among others [4]. In addition to pathogens, mosquitoes harbor diverse microbial communities, including commensals and endosymbionts, collectively termed the microbiota [5]. These microbes play crucial roles in vector survival, fitness, and vector competence [6]. Understanding the assembly and dynamics of mosquito microbial communities is essential for elucidating vector-pathogen interactions and developing novel disease management strategies [7].

Nestedness theory [8] provides a valuable framework for understanding the diverse patterns of interactions between symbionts, commensals, and pathogens within the mosquito microbiota [9]. Nested patterns, characterized by structured interactions among species or taxa subsets, have been extensively studied in various ecological systems [10, 11]. Analyzing nestedness in mosquito microbiota networks can provide insights into the ecological relationships among different taxa and their implications for vector biology [9].

In this study, we investigate the differential nested patterns of *Wolbachia* endosymbionts and the commensal bacterial taxon *Escherichia-Shigella* within the microbial communities of laboratory-reared *Cx. pipiens* f. *molestus* and *Cx. quinquefasciatus*. *Wolbachia*, being an endosymbiont [12], is known to manipulate host reproduction and influence pathogen transmission [13, 14]. It is also known to modulate microbiome structure of insects [15–17]. In laboratory-reared mosquitoes, where environmental conditions are controlled and stable, *Wolbachia* may exhibit a more consistent and structured pattern within the microbiota. Its presence and abundance may be tightly linked to the mosquito host’s physiological state and reproductive biology, leading to a nested pattern within the microbial community. On the other hand, *Escherichia-Shigella* is of interest due to its ability to modulate the mosquito microbiota and influence malaria transmission [7]. Bacteria from the Enterobacteriaceae family are associated with natural microbial communities of mosquito vectors [18]. *Escherichia-Shigella*, as a commensal bacterium [19], may display a different pattern within the microbiota. Its abundance and occurrence could be influenced by various factors such as diet, environmental conditions, and interactions with other microbial taxa [20]. In laboratory-reared mosquitoes, where environmental factors are controlled, *Escherichia-Shigella* may still exhibit dynamic patterns within the microbiota due to its versatile ecological roles and interactions with the host.

Therefore, here we hypothesized that *Wolbachia* endosymbionts would form a nested pattern, reflecting its stable and co-evolved relationship within the host, while *Escherichia-Shigella* may exhibit a less consistent pattern within the microbial community, influenced by environmental and ecological factors. By examining the differential nested patterns of *Wolbachia* endosymbionts and *Escherichia-Shigella* within the mosquito microbiota, we aim to gain insights into the complex interactions shaping vector microbiota assembly. This research may contribute to our understanding of vector-microbiota dynamics and inform the development of novel strategies for controlling mosquito-borne diseases in both public health and veterinary settings.

## Methods

### Ethical statement

The study utilizes two species of laboratory-reared mosquitoes, *Culex pipiens* f. *molestus* and *Culex quinquefasciatus*. While conducted in accordance with ethical standards for animal use in scientific research, it is noted that mosquitoes are not protected under current laws of Lithuania.

### Maintenance of mosquitoes

We analysed P. B. Šivickis parasitology laboratory-reared *Culex quinquefasciatus* and *Cx. pipiens* f. *molestus* mosquitoes. The colonies were maintained as described in Žiegytė et al. study [21]. Briefly, two species of mosquitoes were kept in separate rooms in a nylon netted cage (45 × 45 × 120 cm) under controlled conditions (room temperature 23 ± 1° C; humidity 50–60%; photoperiod 14:10 light: dark). Adult insects were provided with cotton wools saturated with 5% saccharose solution. Larvae were fed with aquarium fish food flakes (“JBL NovoRed“, JBL GmbH & Co. KG, Germany).

### Mosquitoes’ dissection

Mosquito females of *Cx. quinquefasciatus* and *Cx. pipiens* f. *molestus* were haphazardly collected with an insect aspirator from the colonies (*n* = 39 of each species). Before dissection, mosquitoes were euthanised by shaking vigorously to stun them in an insect aspirator. The mosquito wings and legs were removed. The dissection was performed under the binocular stereoscopic microscope. Each mosquito was carefully separated into two segments, the thorax with head and abdomen. The abdomen was placed in a drop of saline, and the midgut of the mosquito was extracted. The midguts were pooled up to 3–4 in sterile microtubes and frozen at -20 °C for microbiota analysis. To prevent contamination of samples, new dissecting needles were used for each pool of dissected insects.

### DNA extraction and 16 S rRNA sequencing

The DNA was extracted from frozen midguts using a Pure Link Microbiome DNA Purification Kit (Invitrogen, Thermo Fisher Scientific, CA, USA). Bound DNA was eluted in 70 µL of elution buffer. Genomic DNA quality (OD260/280 between 1.8 − 2.0) was measured with NanoDrop™ One (Thermo Scientific, Waltham, MA, USA). Sequencing of the 16 S rRNA gene amplicons utilized over 200 ng of DNA at a concentration of 20 ng/µL. The procedure was outsourced to Novogene Bioinformatics Technology Co. (London, UK). DNA libraries were prepared using the NEBNext^®^ Ultra™ II DNA Library Prep Kit from New England Biolabs (MA, USA). Illumina MiSeq sequencing was performed on a single lane, generating 251-base paired-end reads targeting the V4 variable region of the 16 S rRNA gene. Barcoded universal primers (515 F/806R) were employed in the sequencing of mosquito midgut samples, *Cx. quinquefasciatus* (*n* = 13) and *Cx. pipiens* f. *molestus* (*n* = 13). The raw 16 S rRNA sequences obtained from mosquito midguts were deposited at the SRA repository (Bioproject No. PRJNA1114695).

### Identification and removal of contaminants from the sequencing data

Extraction reagent controls were set in which the DNA extraction processes were followed using the same conditions as for the samples but using water as template. DNA amplification was subsequently carried out on the extraction controls under the same conditions applied to the other samples. To statistically identify potential contaminant DNA in the samples intended for 16S rRNA gene sequencing, the ‘decontam’ package [22] was utilized, employing the ‘prevalence’ method. This method defines prevalence as the presence or absence of sequence features across samples and compares the prevalence in actual samples to that in negative controls to detect contaminants. Identified contaminants were then excluded from the dataset prior to further microbiome analysis [22].

### 16 S rRNA sequences processing

The analysis of 16 S rRNA sequences was conducted through the QIIME 2 pipeline (v. 2023.5) [23]. Initial processing involved denoising and merging of sequences within the fastq files, utilizing the DADA2 software [24] as integrated into QIIME 2. Subsequently, amplicon sequence variants (ASVs) were aligned using MAFFT via q2-alignment plugin [25] and employed to construct a phylogeny with FastTree2 via q2-phylogeny [26]. Taxonomic classification of ASVs was performed using a pre-trained classify-sklearn naïve Bayes classifier [27]. This classifier was trained on the SILVA database (release 138) [28], specifically for the V4 region bound by the 515 F/806R primer pair. Taxonomic data tables were then collapsed at the genus level and subjected to filtration to exclude taxa with fewer than 10 total reads and presence in less than 3 samples within each dataset.

### Statistical analysis of microbial diversity and abundance

Alpha and beta diversity metrics were computed through the q2-diversity plugin in QIIME 2 [23]. Shannon diversity index [29], Pielou’s evenness index [30], Faith’s phylogenetic diversity index [31] and observed features [32] were compared between the mosquito using the Kruskal–Wallis test (p < .05) within QIIME 2 [23]. Beta diversity was evaluated using the Bray-Curtis dissimilarity index [33] and compared between groups using the PERMANOVA test (p < .05) also implemented in QIIME 2 [23]. Bacterial variability within the population, known as dispersion, was computed using the ‘betadisper’ function in the Vegan package [34] within R ver. 4.2 [35]. The dispersion between the species was further analyzed using the ANOVA test (*p* < 0.05).

The disparities in taxa abundance between the mosquito species were evaluated using the ANOVA-like differential expression package ‘ALDEx2’ [36] within the R program (ver. 4.2.) [35]. This approach employs a centered log ratio (clr) transformation, utilizing the geometric mean of read counts in each sample to assess relative abundance [37]. Statistical comparisons were conducted using a t-test (*p* ≤ 0.05). Visual representation of shared taxa between different conditions was generated using Venn diagrams, facilitated by an online tool (http://bioinformatics.psb.ugent.be/webtools/Venn/; accessed on 17 April 2023).

### Comparative network analysis of microbiota assembly in mosquitoes

Co-occurrence networks were derived for each experimental condition based on taxonomic profiles, employing the Sparse Correlations for Compositional data (SparCC) approach for correlation matrix calculation [38], in the R ver 4.2 [35]. The analysis included only significant positive correlations (weight > 0.7) or negative correlations (weight < -0.7). Network analysis and visualization were performed using Gephi 0.9.2 software [39]. Topological parameters, encompassing node and edge counts, network diameter, average and weighted degrees, average path length, modularity, number of modules, and average clustering coefficient, were computed to characterize each network.

The Core Association Network (CAN) was utilized to examine common nodes and edges across different networks. The core structures of networks from two mosquito species were identified using the Anuran toolbox [40] with default configurations. This analysis was carried out within the Anaconda Python environment [41].

Microbial networks underwent comparison between conditions using the Network Construction and Comparison for microbiome data (NetCoMi) package [42] in R ver 4.2 [35]. A differential network was generated finding the correlations that vary between identical taxa in two bacterial networks. An association analysis gauged similarities between networks via shared nodes and edges using the same network layout in both groups. Two p-values, P(J ≤ j) and P(J ≥ j), were computed for each Jaccard index, indicating the probability of observing the Jaccard index value equal to or less than, or greater than or equal to, the expected value at random (significance at *p* < 0.05). The similarity between networks was further explored using the Jaccard index, considering various centrality measures, including degree, betweenness, closeness, eigenvector centrality, and hub taxa. This index measures the similarity between nodes with centrality scores above the 75% quartile, ranging from 0 (completely dissimilar) to 1 (identical).

To assess clustering dissimilarity in networks, the adjusted Rand index (ARI) was calculated, with values ranging from − 1 to 1. Positive or negative ARI values indicate higher or lower clustering than random, respectively, with identical clustering having an ARI value of 1 and dissimilar clustering having an ARI value of 0 [42].

To assess the network’s robustness to node removal, the Network Strengths and Weaknesses Analysis (NetSwan) package was utilized [43]. Various node removal strategies, including random, betweenness centrality, degree, and cascading, were executed to gauge network tolerance in terms of connectivity loss. The standard error for connectivity loss was computed, accounting for variability, using a threshold of 0.975. The proportion of nodes removal required to achieve connectivity loss of 80% for each network was evaluated. Bacterial co-occurrence network analysis and visualization were conducted using the igraph package [44].

Furthermore, the robustness of microbial networks to node addition was evaluated by employing the Network analysis and visualization igraph package [44]. Nodes were systematically added in increments ranging from 1 to 1000, and network connectivity was quantified based on the degree metric of the largest connected component (LCC) and average path length. Statistical significance for LCC and average path length was determined using a Wilcoxon signed-rank test, with p-values adjusted using the Benjamini-Hochberg (BH) method to control the false discovery rate. Additionally, bootstrapping was performed to derive confidence intervals for the variables, with significance established at a threshold of *p* < 0.05.

### Local connectivity of Escherichia-Shigella and Wolbachia in the microbial communities of Cx. Quinquefasciatus and cx. Pipiens f. molestus and in silico removal of target taxa

In order to explore the relationship between *Escherichia-Shigella/Wolbachia* and other bacterial members of the microbiota, the *Escherichia-Shigella/Wolbachia* was depicted in connection with all taxa it exhibited positive or negative correlations with, creating *Escherichia-Shigella/Wolbachia* sub-networks. These sub-networks were exported and analysed by comparing interacting partners between the conditions. The analyses were conducted using Gephi software [39] and the online tool of Venn diagrams (http://bioinformatics.psb.ugent.be/webtools/Venn/; accessed on 17 April 2023).

To further investigate the nestedness of *Escherichia-Shigella/Wolbachia* in the networks, we conducted an in-silico experiment where *Escherichia-Shigella/Wolbachia* was removed from the networks to observe its effect on node centrality distribution and network robustness. The comparisons of the networks with and without *Escherichia-Shigella/Wolbachia* in *Cx. quinquefasciatus* and *Cx. pipiens* f. *molestus* were performed following the procedures as described above.

### Keystone taxa identification

The keystone taxa were identified based on three established criteria, as outlined in previous studies [45, 46]: (i) ubiquitous presence, (ii) high eigenvector centrality (≥ 0.75), and (iii) high relative abundance (clr value exceeding the average).

## Results

### Differential structure of bacterial microbiota in Culex pipiens f. molestus and Culex quinquefasciatus

**Fig. 1:**
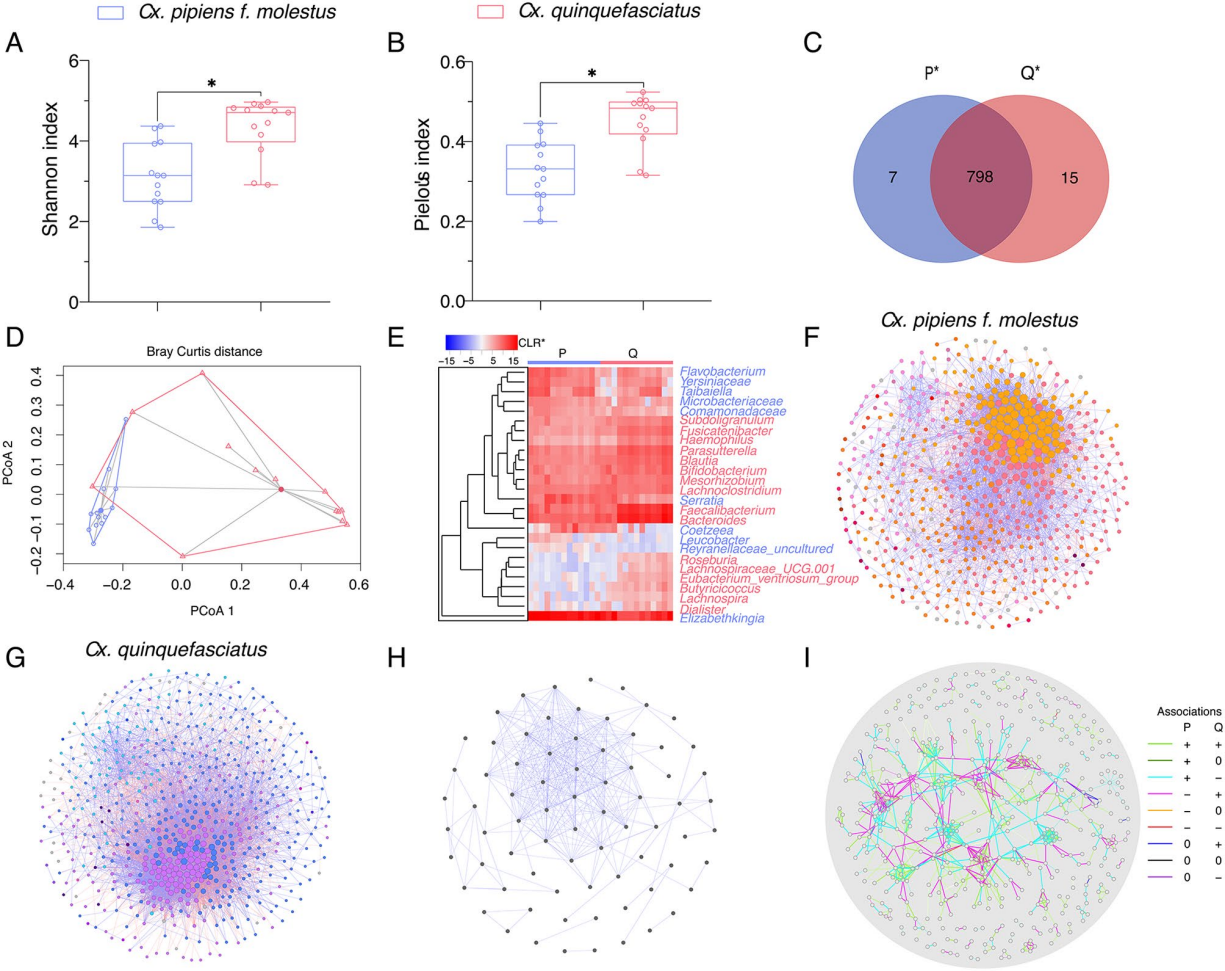
Differences in mosquito microbiota diversity and community assembly between *Culex pipiens* f. *molestus* and *Culex quinquefasciatus.* (**A**) Shannon diversity and (**B**) Pielou’s evenness indices showed significant differences between microbiota of *Cx. pipiens* f. *molestus* and *Cx. quinquefasciatus*. (**C**) Venn diagram showing the number of bacterial taxa that are shared or unique among the networks of two mosquito species. *p < 0.05 (**D**) Beta diversity of mosquito microbiota of two species represented in PCoA plot obtained by Betadisper function. There are significant differences in dispersions (variances) (ANOVA, *p* < 0.01). (**E**) Heatmap representing the abundance (expressed as *Centered Log-Ratio) of the 10 taxa whose abundance was higher *in Cx. pipiens* f. *molestus* group and 16 taxa whose abundance was higher in *Cx. quinquefasciatus*. (**F**, **G**) Bacterial co-occurrence networks were inferred from 16SrRNA sequences obtained from laboratory reared mosquitoes of two species (**F**) *Cx. pipiens* f. *molestus* and (**G**) *Cx. quinquefasciatus* (SparCC > 0.5 or < -0.5). Nodes correspond to taxa (family or genus level). The colours of nodes specify modules in which taxa occur. The size of nodes is related to their eigenvector centrality, the bigger the node, the higher eigenvector centrality value it has. Positive (purple) and negative (coral) correlations are shown by the colour of the edges. (**H**) Core Association Network (CAN) (SparCC > 0.75 or < -0.75). Positive correlations are shown by purple edges. Nodes correspond to taxa. (**I**) Differential network between *Cx. pipiens* f. *molestus* and *Cx. quinquefasciatus* natural networks illustrating the correlations that vary between identical taxa in two bacterial networks. Grey nodes represent taxa, and edges represent differential associations between taxa. P^*^ - *Cx. pipiens* f. *molestus*; Q^*^ - *Cx. quinquefasciatus*; CLR* - Centered log ratio

After statistically identifying and removing DNA features classified as contaminants (Table S1), the bacterial community composition and diversity of mosquito microbiota were analyzed using 16 S rRNA gene profiling, followed by a comparative network analysis to assess microbial sample similarity between two mosquito species and identify species-specific patterns of *Escherichia*-*Shigella* and *Wolbachia* nestedness. The comparison of microbial composition between *Culex pipiens* f. *molestus* and *Culex quinquefasciatus* species showed differences in alpha (i.e., Shannon diversity index, Pielou’s evenness index, Faith’s phylogenetic diversity index, and observed features) and beta diversity (Bray Curtis index and beta dispersion) metrics. Significantly higher Shannon and Pielou’s indices were observed (*p* < 0.05; Fig. 1A, B) in the *Cx. quinquefasciatus* group compared to *Cx. pipiens* f. *molestus*, while the differences in Faith’s phylogenetic diversity and observed features did not have statistical significance between the groups. However, the compositional analysis revealed that two mosquito species shared most of the existing bacteria in the microbiota (97% in total of 820 taxa; Fig. 1C), while 7 and 15 were unique to *Cx. pipiens* f. *molestus* and *Cx. quinquefasciatus*, respectively (Table S2). Comparison of Bray-Curtis dissimilarity indices and beta dispersion between the species showed significant differences (PERMANOVA, F = 19.36, *p* = 0.001; ANOVA, F = 10.023, *p* = 0.001, respectively; Fig. 1D). Differential relative abundance analysis identified 10 taxa, which had significantly higher abundance in the microbiota of *Cx. pipiens* f. *molestus*, and 16 taxa more abundant in *Cx. quinquefasciatus* (Welch t-test, *p* < 0.05; Fig. 1E).

Bacteria co-occurrence networks were inferred to compare microbiota assembly between two mosquito species (Fig. 1F, G). Visual inspection of the networks and their features revealed some differences between the species (Fig. 1F, G; Table 1). The network of *Cx. quinquefasciatus* consists of a higher number of edges compared to the *Cx. pipiens* f. *molestus*, while the number of nodes is similar between the species (Fig. 1F, G; Table 1). This relates to a higher value of average degree in *Cx. quinquefasciatus* group compared to the other mosquito species as this topological parameter indicates an average number of edges connected to a node (Table 1). However, the weighted degree value is greater on *Cx. pipiens* f. *molestus* species referring to stronger correlations in the network. Although the number of modules is similar in both groups, the lower value of modularity suggests higher interconnectedness between modules in *Cx. pipiens* f. *molestus* network compared to *Cx. quinquefasciatus* (Table 1). Core Association Network (CAN) analysis revealed 74 core associated nodes and 337 positive edges between the groups of *Cx. pipiens* f. *molestus* and *Cx. quinquefasciatus* (Fig. 1H). Furthermore, network dissimilarity analysis revealed differential associations between taxa in the groups (Fig. 1I).

**Table 1:** Network features

Network features	Cx. pipiens f. molestus	Cx. quinquefsciatus
Nodes	592 (805)	642 (813)
Edges	6577	10,514
Positive	4185 (64%)	5783 (55%)
Negative	2392 (36%)	4731 (45%)
Network diameter	9	7
Average degree	22.22	32.754
Weighted degree	4.219	2.571
Average path length	3.14	2.871
Modularity	1.392	2.89
Number of modules	45	40
Average clustering coefficient	0.454	0.489

A statistical network comparison analysis was performed to evaluate the differences in local centrality measures between the groups (Table 2). Jaccard index values for a degree, hub taxa, betweenness, closeness, and eigenvector centrality between the two mosquito species varied from 0.4 to 0.5, which were significantly higher than expected by random (P (≥ Jacc) < 0.05) suggesting a moderate degree of similarity.

**Table 2:** Jaccard index for *Cx. Pipiens* f. *molestus* and *cx. Quinquefasciatus* networks

Local centrality measures	Cx. pipiens f. molestus vs. Cx. quinquefasciatus		
	Jacc^a^	*P*( < = Jacc)	*P*( > = Jacc)
Degree	0.458	0.999994	0.000010*
Betweenness centr.	0.459	0.999995	0.000008*
Closeness centr.	0.470	0.999999	0.000002*
Eigenvec. centr.	0.419	0.998997	0.001495*
Hub taxa	0.507	1.000000	0.000000*

^a^Jaccard index

^*^p < 0.05

### *Placement of* Escherichia-Shigella *and* Wolbachia *across equivalent modules in* Culex pipiens f. molestus *and* Culex quinquefasciatus *Microbiota networks*

**Fig. 2:**
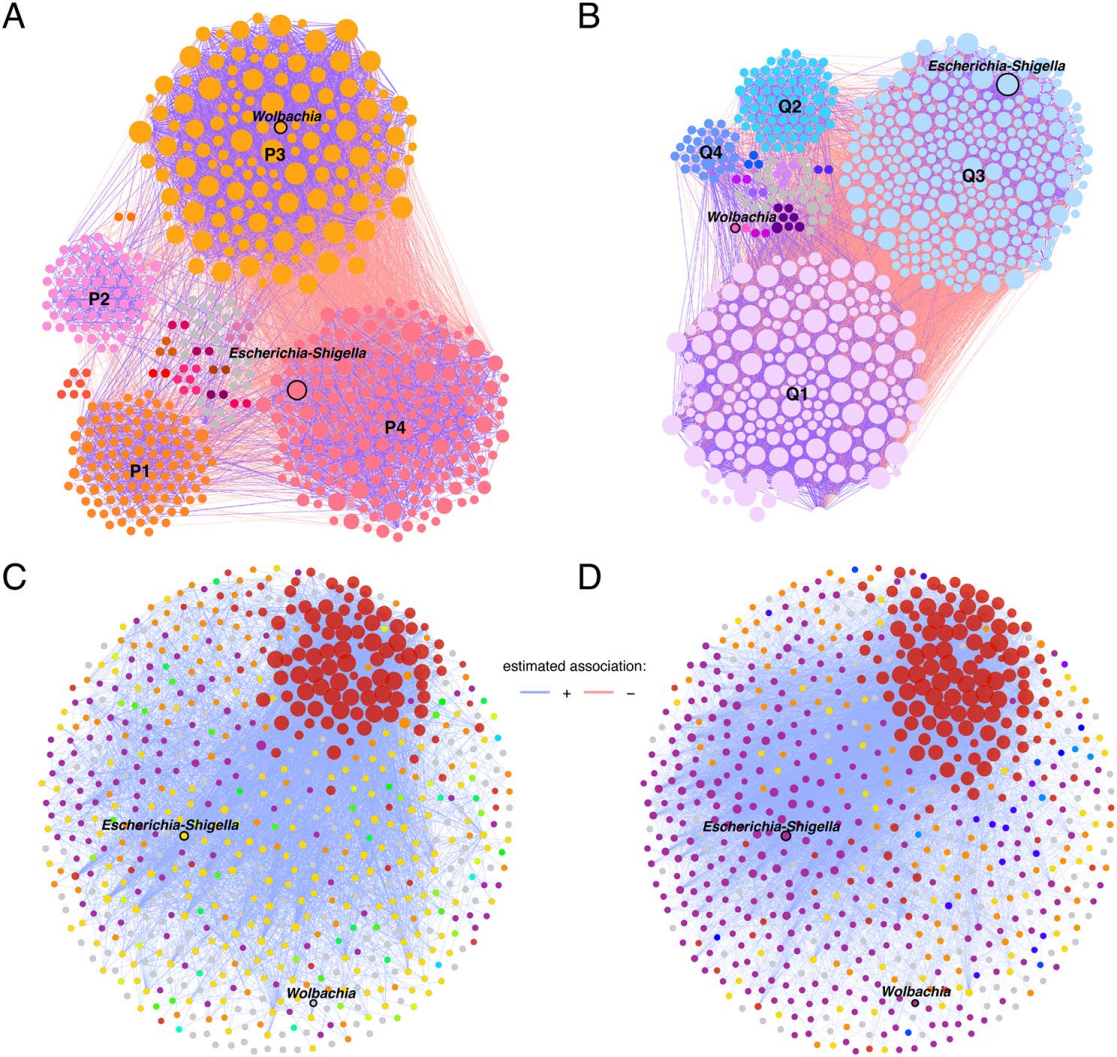
Differences in mosquito bacterial modules between *Culex pipiens* f. *molestus* and *Culex quinquefasciatus*. (**A**, **B**) Bacterial co-occurrence networks of (**A**) *Cx. pipiens* f. *molestus* and (**B**) *Cx. quinquefasciatus* divided by modules. Node colours are based on modularity class metric, each module is represented by a different colour. Grey colored nodes represents single node modules. The size of nodes is related to their eigenvector centrality, the bigger the node, the higher eigenvector centrality value it has. Positive (purple) or negative (coral) correlations are shown by the colour of the edges. Bacterial taxa (family or genus level) with at least one connection are symbolized by nodes, whilst connected edges represent correlations between them (SparCC ≥ 0.5 or ≤ -0.5). (**C**, **D**) Co-occurrence networks in the same layout were extrapolated from the microbiota of (**C**) *Cx. pipiens* f. *molestus* and (**D**) *Cx. quinquefasciatus*. Bacterial taxa (family or genus level) with at least one connection are symbolized by nodes, whilst connected edges represent a significant correlation between them (SparCC ≥ 0.75 or ≤ -0.75). Node colours are based on determined clusters and sized according to the node’s eigenvector centrality. Positive (purple) or negative (red) correlations are shown by the colour of the edges

The networks of *Cx. pipiens* f. *molestus* and *Cx. quinquefasciatus* group to 4 major modules composed of 89% and 91% of nodes of a whole network, respectively. The biggest modules of *Cx. pipiens* f. *molestus* (P4 – the total of 208 nodes; Fig. 2A) and *Cx. quinquefasciatus* (Q3 – the total of 294 nodes; Fig. 2B) consists of the highest number of unique nodes, 19% and 21%, respectively. The comparison of taxa in these modules showed that 93 (22.7%; Table 3) nodes are shared between the *Cx. pipiens* f. *molestus* and *Cx. quinquefasciatus.* Based on the percentage of taxa shared between the modules, Q3 and P4 exhibit a moderate level of equivalence. In addition, the modules Q3 and P4 accommodate the commensal bacterial taxon *Escherichia-Shigella* (Fig. 2A, B). However, the module Q3 (294 nodes) of *Cx. quinquefasciatus* could be considered more equivalent to P3 (105 nodes) module of *Cx. pipiens* f. *molestus* based on the percentage of shared taxa of 24.3% (Table 3).

**Table 3:** The percentage of taxa shared between different modules

	P1	P2	P3	P4
Q1	38%	1.9%	4.2%	9.9%
Q2	0.9%	30.1%	2.3%	8.0%
Q3	5.8%	10.3%	24.3%	22.7%
Q4	1.2%	2.1%	2.3%	6.3%

Notably, in *Cx. pipiens* f. *molestus*, *Wolbachia* was found in module P3 (Fig. 2A), while it formed a distinctive module in *Cx. quinquefasciatus* (Fig. 2B). The highest equivalence between the species was estimated between the second major modules consisting of 143 (P1) and 191 (Q1) nodes. Between the modules, 92 nodes are shared (38%; Table 3). Modules P2 (71 nodes) and Q2 (76 nodes) are similar in size and share 34 nodes (30.1%) (Table 3). The network of *Cx. quinquefasciatus* has a smaller module Q4 of 28 nodes, which does not have an equivalent in the network of *Cx. pipiens* f. *molestus*, however, 14 nodes are shared with P4 module (6.3%) (Table 3). Visual inspection of interactions inside modules shows that positive edges are dominant in both species. The same pattern of interactions was seen in the networks of *Cx. pipiens* f. *molestus* and *Cx. quinquefasciatus* where the two biggest modules of a network had mostly negative edges between each other (Fig. 2A, B). An estimated association analysis was performed to further analyse microbial associations in the networks (Fig. 2C, D). In the networks of the same layout the clusters and modules are seen to be different between the species. However, one cluster is similar between the networks (Fig. 2C, D, red cluster), consisting of mostly shared taxa with higher eigenvector centrality values. The adjusted Rand index (ARI) was 0.151 (*p* = 0.000), showing some clustering similarities between the networks.

**Fig. 3:**
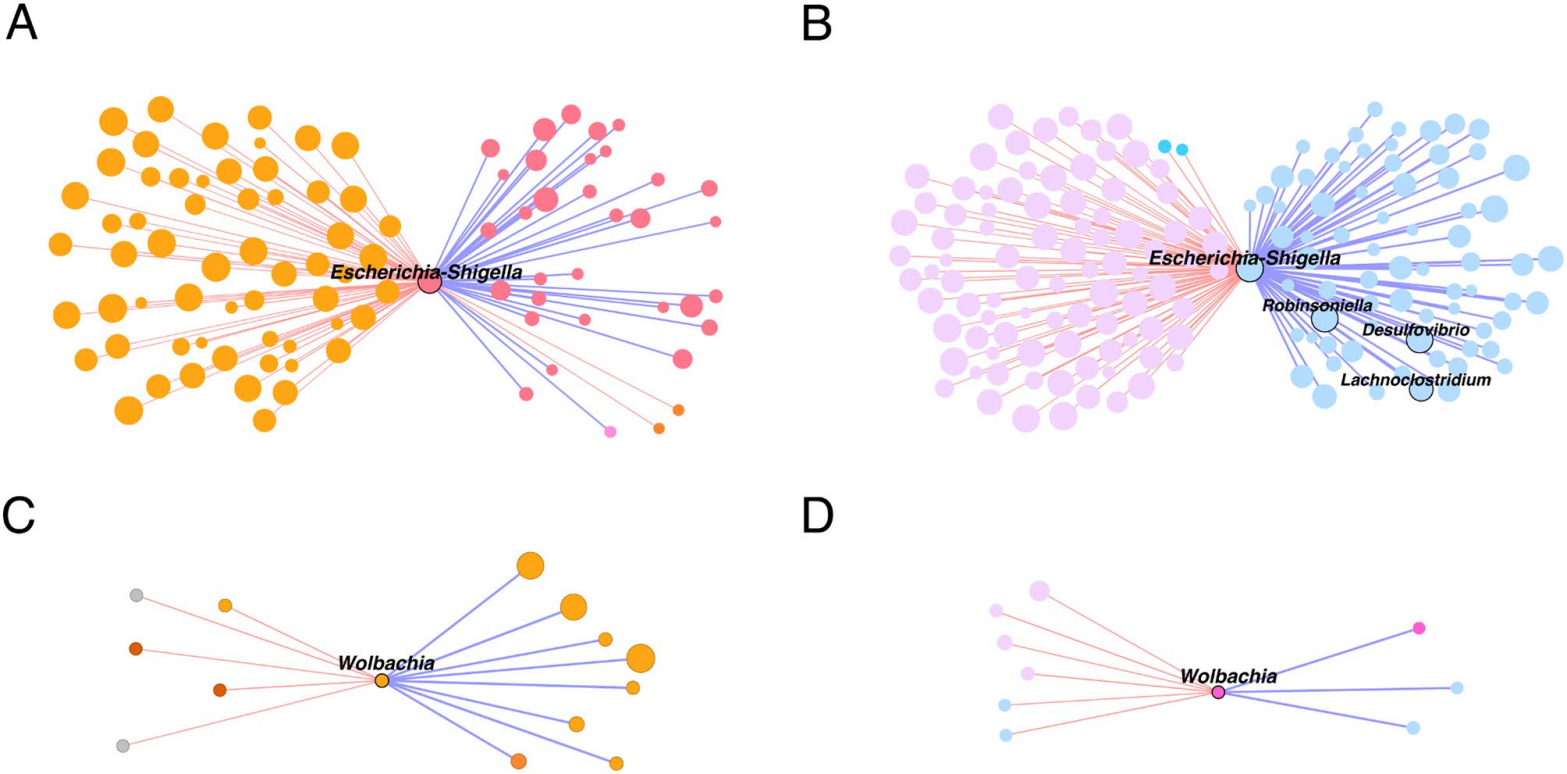
Differences in the local connectivity of *Escherichia-Shigella* and *Wolbachia* between *Culex pipiens* f. *molestus* and *Culex quinquefasciatus* microbiota. (**A**, **B**) Sub-networks of the local connectivity of *Escherichia-Shigella* and (**C**, **D**) *Wolbachia* were extracted from (**A**, **C**) *Cx. pipiens* f. *molestus* and (**B**, **D**) *Cx. quinquefasciatus* natural networks. The size of nodes is related to their eigenvector centrality, the bigger the node, the higher eigenvector centrality value it has. Positive (purple) or negative (coral) correlations are shown by the colour of the edges. Bacterial taxa (family or genus level) with at least one connection are symbolized by nodes, whilst connected edges represent correlations between them (SparCC ≥ 0.5 or ≤ -0.5)

Based on taxa ubiquitousness, relative abundance and eigenvector centrality (> 0.7), keystone taxa were identified in the networks of *Cx. pipiens* f. *molestus* and *Cx. quinquefasciatus*. One keystone taxon, *Escherichia-Shigella*, was identified in the microbiota of *Cx. pipiens* f. *molestus* (P4 module; Fig. 2A). This taxon has positive correlations with other taxa from the P4 module (Figs. 2A and 3A). However, only negative correlations are formed with taxa of the P3 module (Fig. 3A). Bacteria *Escherichia*-*Shigella*, as a keystone taxon, was also identified in the microbiota of *Cx. quinquefasciatus* alongside *Lachnoclostridium*,* Robinsoniella*,* Desulfovibrio*,* Muribaculum.* All keystones except *Muribaculum* are part of the Q3 module (Fig. 2B). The keystones have positive correlations between each other and with other taxa from the Q3 modules; negative correlations are formed with taxa from the Q1 module (Figs. 2B and 3B). The placement of the taxon *Escherichia*-*Shigella* could be considered equivalent inside the networks of two mosquito species. It has correlations with 93 (Fig. 3A) and 161 (Fig. 3B) nodes in the networks of *Cx. pipiens* f. *molestus* and *Cx. quinquefasciatus*, respectively. The comparison of interacting bacteria showed 55 shared taxa between the species.

We determined the associations of common symbiont *Wolbachia* in the networks of both groups. The symbiont was prevalent in all samples of the study, which abundance did not differ significantly between the mosquito species. The number of ASVs classified as *Wolbachia* ranged from 3 to 512 in *Cx. pipiens* f. *molestus* samples and from 31 to 892 in *Cx. quinquefasciatus* samples. *Wolbachia* belonging to the P3 module of *Cx. pipiens* f. *molestus* has positive interactions within the P3 module and mostly negative interactions with nodes from other modules (Figs. 2A and 3C). *Wolbachia* in the network of *Cx. quinquefasciatus* forms a distinctive module together with bacteria SB-5 belonging to phylum Bacteroidota. It forms mostly negative interactions within the network with nodes from modules Q3 and Q1. The placement of *Wolbachia* inside the networks is not equivalent. The symbiont has 13 (Fig. 3C) and 9 (Fig. 3D) correlating nodes within the networks of *Cx. pipiens* f. *molestus* and *Cx. quinquefasciatus*, respectively. None of the interacting taxa are shared between the species.

While *Escherichia-Shigella* and *Wolbachia* do not cluster together in the network of *Cx. pipiens* f. *molestus* (Fig. 2C), in the network of *Cx. quinquefasciatus* (Fig. 2D) mentioned bacteria belong to the same cluster.

### *Effect of* Escherichia-Shigella *and* Wolbachia *on network robustness in* Culex pipiens f. molestus *and* Culex quinquefasciatus

An in-silico experiment was carried out to assess the potential influence of *Escherichia-Shigella* and *Wolbachia* on the microbial assembly and structure of *Cx. pipiens* f. *molestus* and *Cx. quinquefasciatus.* Only minor changes were recorded in the networks’ features after removing either *Escherichia-Shigella* or *Wolbachia* (Table S3). After the removal of target taxa, an increase in the number of modules was recorded in all networks (Table S3). The clusters with which *Wolbachia* associated changed after the removal of *Escherichia-Shigella* in the networks of *Cx. pipiens* f. *molestus* (Figure S1A) and *Cx. quinquefasciatus* (Figure S1B). However, the removal of *Wolbachia* from the networks did not change the clusters of *Escherichia-Shigella* (Fig. 2C, D; Figure S1C, D). Local centrality measures of natural networks were compared to the ones with removed taxon *Escherichia-Shigella* or *Wolbachia* using the Jaccard index (Table S4). All measures were significantly higher than expected by random with high Jaccard index values (Table S4). Differential network analysis did not detect any differentially associated taxa. These findings suggest that target taxa removal did not greatly affect the node centrality traits of a network.

**Fig. 4:**
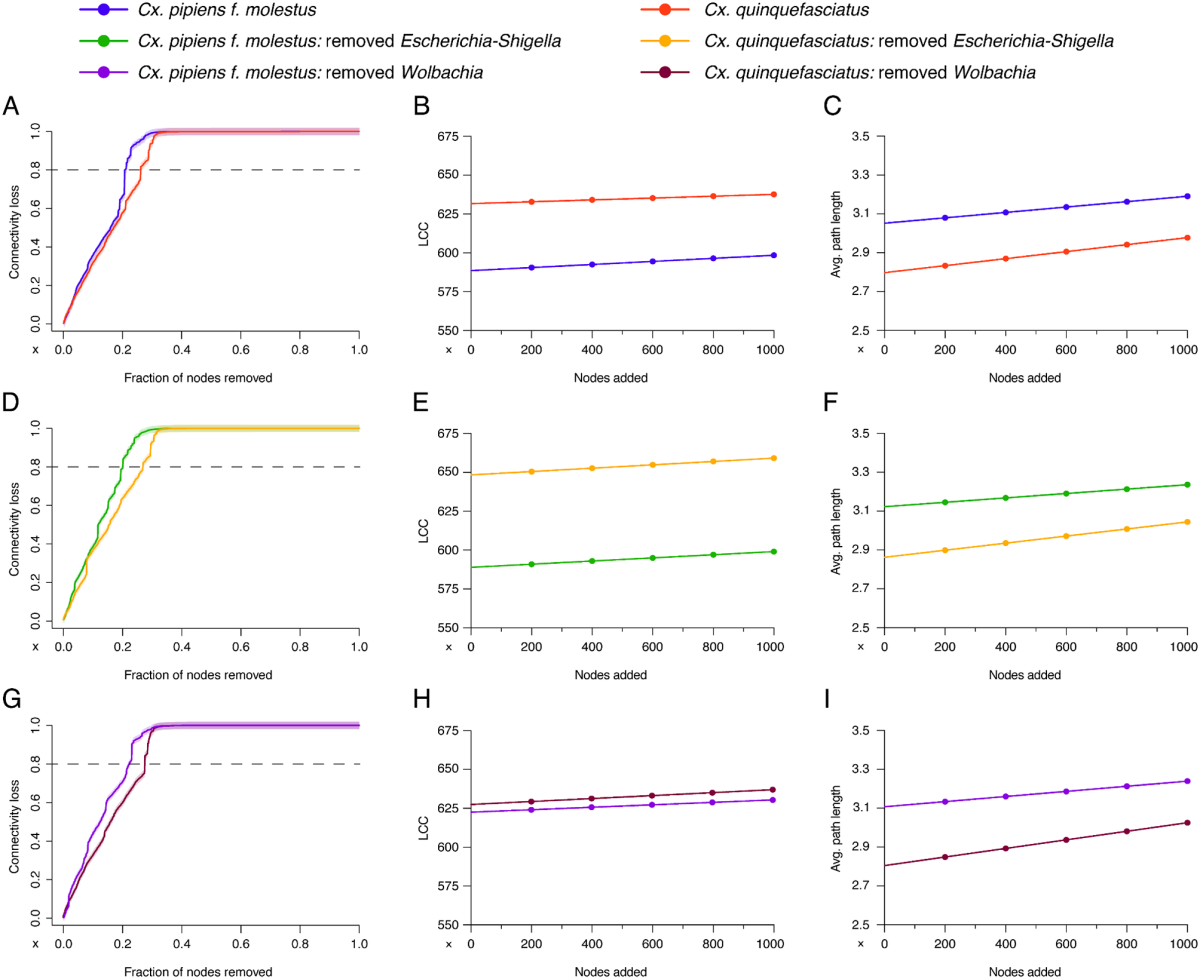
Differences in the microbial network response to node removal and addition between *Culex pipiens* f. *molestus* and *Culex quinquefasciatus*. (**A**, **D**, **G**) The resistance to cascading attack was measured and compared between *Cx. pipiens* f. *molestus* and *Cx. quinquefasciatus* (**A**) natural networks, (**D**) networks without *Escherichia-Shigella* or (**G**) *Wolbachia*. The robustness to nodes addition (from 0 to 1000) based on the size of the largest connected component (LCC) (**B**, **E**, **H**) and average path length (avg. path length) (**C**, **F**, **I**) was measured and compared between *Cx. pipiens* f. *molestus* and *Cx. quinquefasciatus* natural networks (**B**, **C**), networks without *Escherichia-Shigella* (**E**, **F**) and *Wolbachia* (**H**, **I**). Different curve colours represent different groups

To further analyse the differences between the species and how target taxa removal or addition affects the network, we performed multiple comparisons of network robustness after node removal and addition (Fig. 4, Figure S2, and Figure S3). Node removal by cascading attack had the most significant impact on connectivity in all networks (Figure S2). The difference in network resistance to node removal was evaluated by calculating the delta value of the percentage of nodes removed. The highest delta value between the networks of *Cx. pipiens* f. *molestus* and *Cx. quinquefasciatus* was recorded at 80% of connectivity loss (Fig. 4A, D, G). The delta between the natural networks was 5% (Fig. 4A). The target taxa removal from the networks caused a slight increase in the delta value of robustness to 7% and 6% of networks without *Escherichia-Shigella* (Fig. 4D) and *Wolbachia* (Fig. 4G), respectively. Node addition did not greatly affect the network robustness based on the largest connected component ((LCC); Fig. 4B, E, H) and the average path length (Fig. 4C, F, I) of neither of the species (Figure S3). However, the network of *Cx. quinquefasciatus* initially has a bigger LCC and shorter average path length compared to *Cx. pipiens* f. *molestus* in all comparisons (Fig. 4). The difference in the size of the LCC between the species has increased between the networks without *Escherichia-Shigella* (Fig. 4E) and decreased between the networks without *Wolbachia* (Fig. 4H) compared to the natural network (Figure S3). No change in average path length was recorded between *Cx. pipiens* f. *molestus* and *Cx. quinquefasciatus* after removing target taxa from the networks (Fig. 4C, F, I).

The robustness of natural networks was compared to that of the ones without the target taxa. In silico removal of *Escherichia-Shigella* and *Wolbachia* did not have the same effects on the network robustness of *Cx. pipiens* f. *molestus* and *Cx. quinquefasciatus* (Figure S3). The difference in connectivity loss between the natural networks and without the target taxa was only noticed in *Cx. pipiens* f. *molestus* group (Figure S3A; S3D; S3G; S3J). Cascading removal of nodes showed deltas of -4% for the network without *Escherichia-Shigella* and − 2% for the network without *Wolbachia* (Figure S3A, D). There was no change in the size of LCC in the network of *Cx. pipiens* f. *molestus* without *Escherichia-Shigella* (Figure S3B) and *Cx. quinquefasciatus* without *Wolbachia* (Figure S3K). However, LCC has increased in the network of *Cx. pipiens* f. *molestus* without *Wolbachia* (Figure S3E). and *Cx. quinquefasciatus* without *Escherichia-Shigella* (Figure S3H). The average path length has slightly increased after taxa removal in all the networks (Figure S3C, F, I, L).

## Discussion

Our research uncovers complex ecological interactions and patterns among commensal and endosymbiotic microbes. These microbes are crucial for synthesizing essential nutrients and significantly influence mosquito health and ability to transmit diseases, aligning with findings from Minard et al. [47] and Douglas [48] that highlight the essential role of microbial communities in insect nutritional ecology and overall fitness.

There is limited published literature on the specific microbiota network complexity of *Cx. pipiens* f. *molestus* and *Cx. quinquefasciatus*. Our results revealed a moderate degree of similarity between the microbiota of the two mosquito species. This similarity may be attributed to their taxonomic closeness and shared ecological niches, echoing the results of other studies, showing that closely related mosquito species tend to harbor similar microbial communities due to ecological and evolutionary constraints [20]. These findings suggest that a stable nested pattern of *Wolbachia* endosymbionts might be observed in both species, while some differences might appear in the structure surrounding the commensal bacteria *Escherichia-Shigella*.

First, we compared the microbial diversity between the species, which showed a high number of shared taxa between *Culex pipiens* f. *molestus* and *Culex quinquefasciatus*, highlighting the influence of the ecological setting on microbiota composition and diversity. The environment in which both mosquito species were raised was controlled and stable, which likely led to the high similarity in their microbiota diversity. This observation aligns with the findings of Muturi et al. [49], who reported that environmental factors play a pivotal role in shaping the mosquito microbiota, thereby affecting the host’s vectorial capacity. The shared microbiota between these species suggests common ecological pressures and evolutionary histories that influence their microbial community structures [50]. Our results show that variability of microbial composition is different between the species, *Cx. quinquefasciatus* having more heterogenous communities compared to *Cx. pipiens* f. *molestus*. Lower beta dispersion in *Cx. pipiens* f. *molestus* could imply that the community structure is more stable and less influenced by disturbances. These findings are supported by the robustness analysis showing *Cx. pipiens* f. *molestus* network being more resilient compared to *Cx. quinquefasciatus* (Fig. 4).

While both networks exhibit a degree of complexity, *Cx. quinquefasciatus* has a network characterized by a higher number of edges, suggesting more interactions among microbial taxa (Fig. 2A, B; Table 1). Despite being reared in similar settings, the networks still retain some distinct characteristics influenced by host-specific factors and microbial dynamics. The structural similarities in their microbial networks underscore the potential for common microbial interaction patterns across different mosquito species [20, 51].

Bacterial co-occurrence networks revealed a structured pattern of interactions between commensal bacteria *Escherichia-Shigella* and 55 taxa present in the microbiota of *Cx. pipiens* f. *molestus* and *Cx. quinquefasciatus*. The commensal was identified as a keystone taxon in both species, which points to its critical role within the microbial community (Fig. 3A, B). The keystones likely facilitate essential ecological functions and interactions within their networks, similar to findings in other systems where keystone species contribute disproportionately to community structure and function [52]. Enterobacteriaceae is an important contributor to normal mosquito microbiota, commonly found in larval and adult mosquitoes of different genera such as *Culex*, *Aedes*, *Anopheles* etc [53, 54]. The nature of commensals’ assembly is driven by nutrient (carbon source) availability, with Enterobacteriaceae being one of the fastest growing bacteria [55].

We did not observe a consistent nested pattern of *Wolbachia*, which goes against our hypothesis. *Wolbachia*’s position in the network appears less central, with fewer interactions compared to *Escherichia* (Fig. 3). *Wolbachia*, present in over 60% of insect species, is considered highly important due to its significant effects on mosquito lifespan and immune response. It enhances the mosquito’s defense mechanisms and may outcompete pathogens for cellular resources, inhibiting the transmission of various pathogens [2]. Rasgon and Scott [56] demonstrated that a single *Wolbachia* strain infects *Cx. pipiens*, with infection frequencies near fixation across all populations sampled over two years. Their findings suggest that *Wolbachia* has the potential to invade vector populations and could be leveraged in strategies to reduce vector-borne diseases. There is limited information on how *Wolbachia* impacts the microbiota structure of mosquitoes. Lee et al. [57] demonstrated that *Wolbachia* tends to have antagonistic relationships with other bacteria in the host’s microbial community. A similar pattern has been observed in other insect species, where *Wolbachia*-infected individuals exhibit reduced overall microbial diversity compared to non-infected counterparts [15–17]. The nuanced role of *Wolbachia* as a less interconnected taxon in our study within *Culex* mosquitoes suggests a specialized function within the mosquito microbiota, potentially related to its pathogen-blocking capabilities and influence on host reproductive biology, as documented in previous studies [2, 58]. Although we did not perform strain-specific analyses to determine if the two mosquito species carry identical *Wolbachia* strains, both species were reared under the same controlled laboratory conditions, making it unlikely that they harbor different *Wolbachia* strains. Nonetheless, future studies should incorporate strain typing methods to definitively confirm the similarity of *Wolbachia* strains between the mosquito species.

An in-silico experiment was conducted in this study to understand the impact of removing *Escherichia-Shigella* and *Wolbachia* on the microbial community structure of *Cx. pipiens* f. *molestus* and *Cx. quinquefasciatus*. The results showed only minor changes in network features upon removing these taxa. However, an increase in network modules was observed, suggesting a certain role in microbial network assembly. An increase in the number of modules, with a mostly consistent number of nodes and edges in the network representing associations within the community, indicates an abundance of weaker, interchangeable interactions. This suggests that the network is more resilient and capable of withstanding disturbances in both mosquito species. Such behavior in the interactions within the network was noted in the study by the Coyte et al. (2021) [59]. The centrality measures, indicative of network connectivity and importance, remained significantly high even after taxa removal, indicating the robustness of the microbial communities against the loss of these specific taxa. This finding suggests that certain taxa may have broader ecological roles or competitive advantages within the microbiota, impacting network robustness and resilience [52, 60].

## Conclusions

This study provides a comprehensive analysis of the microbial communities within two mosquito species, *Culex pipiens* f. *molestus* and *Culex quinquefasciatus*, with a specific focus on the differential nested patterns of *Wolbachia* endosymbionts and the commensal bacterial taxon *Escherichia*-*Shigella*. The high similarity in microbiota composition between *Cx. pipiens* f. *molestus* and *Cx. quinquefasciatus* can be attributed to the controlled and stable laboratory conditions under which they were reared. This environmental consistency likely facilitated the development of similar microbial communities, reflecting common ecological pressures and evolutionary histories. However, despite this overall similarity, the beta diversity metrics and network analyses indicated distinct patterns of microbial interactions within each species.

*Escherichia-Shigella* was identified as a keystone taxon in both mosquito species, highlighting its critical role within the microbial community. Its interactions with a substantial number of taxa suggest it plays a significant role in shaping the microbial network structure. Conversely, *Wolbachia* did not exhibit the expected stable nested pattern and showed fewer interactions within the microbial networks. This nuanced role of *Wolbachia* suggests it may function more as a specialized entity within the microbiota, influencing specific aspects of mosquito physiology and pathogen transmission.

The differential nested patterns of *Escherichia-Shigella* and *Wolbachia* provide valuable insights into the assembly and dynamics of mosquito microbial communities. Understanding these interactions is crucial for developing innovative strategies to control mosquito-borne diseases. Future studies should explore the mechanisms driving these interactions and their implications for vector competence and disease transmission, particularly in natural environments where mosquito-microbiota interactions are influenced by more variable and complex ecological factors.

## Electronic supplementary material

Below is the link to the electronic supplementary material.

Supplementary Material 1

Supplementary Material 2

Supplementary Material 3

Supplementary Material 4

Supplementary Material 5

Supplementary Material 6

Supplementary Material 7

Supplementary Material 8

## Data Availability

The datasets generated and analysed during the current study are available in the SRA repository (Bioproject No. PRJNA1114695), https://www.ncbi.nlm.nih.gov/sra.

## Abbreviations

Avg: Average
ARI: Adjusted Rand Index
CAN: The Core Association Network
F: Form
LCC: Largest Connected Component
SparCC: The Sparse Correlations for Compositional data

## References

[1] Caminade C, McIntyre KM, Jones AE. Impact of recent and future climate change on vector-borne diseases. Ann N Y Acad Sci janv. 2019;1436(1):157–73. 10.1111/nyas.13950 PMC6378404 30120891

[2] Moreira LA, Iturbe-Ormaetxe I, Jeffery JA, Lu G, Pyke AT, Hedges LM, et al. A Wolbachia symbiont in Aedes aegypti limits infection with dengue, Chikungunya, and Plasmodium. Cell 24 déc. 2009;139(7):1268–78. 10.1016/j.cell.2009.11.042 20064373

[3] Simón F, González-Miguel J, Diosdado A, Gómez PJ, Morchón R, Kartashev V. The complexity of zoonotic Filariasis Episystem and its consequences: a multidisciplinary view. Biomed Res Int. 2017;2017:6436130. 28642878 10.1155/2017/6436130 PMC5469992

[4] Huang Y-JS, Higgs S, Vanlandingham DL. Emergence and re-emergence of mosquito-borne arboviruses. Curr Opin Virol févr. 2019;34:104–9. 10.1016/j.coviro.2019.01.001 30743191

[5] El-Sayed A, Aleya L, Kamel M. Microbiota’s role in health and diseases. Environ Sci Pollut Res Int Juill. 2021;28(28):36967–83. 10.1007/s11356-021-14593-z PMC8155182 34043164

[6] Romoli O, Schönbeck JC, Hapfelmeier S, Gendrin M. Production of germ-free mosquitoes via transient colonisation allows stage-specific investigation of host-microbiota interactions. Nat Commun. 11 févr. 2021;12(1):942. 10.1038/s41467-021-21195-3 PMC7878806 33574256

[7] Aželytė J, Wu-Chuang A, Žiegytė R, Platonova E, Mateos-Hernandez L, Maye J, et al. Anti-microbiota vaccine reduces avian malaria infection within mosquito vectors. Front Immunol. 2022;13:841835. 35309317 10.3389/fimmu.2022.841835 PMC8928750

[8] Ren Z, Li H, Luo W. Unraveling the mystery of antibiotic resistance genes in green and red Antarctic snow. Sci Total Environ 10 mars. 2024;915:170148. 10.1016/j.scitotenv.2024.170148 38246373

[9] Seabourn PS, Weber DE, Spafford H, Medeiros MCI. Aedes albopictus microbiome derives from environmental sources and partitions across distinct host tissues. Microbiologyopen juin. 2023;12(3):e1364. 10.1002/mbo3.1364 PMC10261752 37379424

[10] Cobo-López S, Gupta VK, Sung J, Guimerà R, Sales-Pardo M. Stochastic block models reveal a robust nested pattern in healthy human gut microbiomes. PNAS Nexus Juill. 2022;1(3):pgac055. 10.1093/pnasnexus/pgac055 PMC9896942 36741465

[11] Song C, Rohr RP, Saavedra S. Why are some plant-pollinator networks more nested than others? J Anim Ecol Oct. 2017;86(6):1417–24. 10.1111/1365-2656.12749 28833083

[12] Werren JH, Baldo L, Clark ME. Wolbachia: master manipulators of invertebrate biology. Nat Rev Microbiol oct. 2008;6(10):741–51. 10.1038/nrmicro1969 18794912

[13] Johnson KN. The impact of Wolbachia on Virus infection in mosquitoes. Viruses 4 nov. 2015;7(11):5705–17. 10.3390/v7112903 PMC4664976 26556361

[14] Torres R, Hernandez E, Flores V, Ramirez JL, Joyce AL. Wolbachia in mosquitoes from the Central Valley of California, USA. Parasit Vectors 10 nov. 2020;13(1):558. 10.1186/s13071-020-04429-z PMC7653878 33168082

[15] Simhadri RK, Fast EM, Guo R, Schultz MJ, Vaisman N, Ortiz L, et al. The gut commensal microbiome of Drosophila melanogaster is modified by the Endosymbiont *Wolbachia*. mSphere. 2017;2:e00287–17. 28932814 10.1128/mSphere.00287-17 PMC5597968

[16] Detcharoen M, Jiggins FM, Schlick-Steiner BC, Steiner FM. Wolbachia endosymbiotic bacteria alter the gut microbiome in the fly Drosophila Nigrosparsa. J Invertebr Pathol. 2023;198:107915. 36958642 10.1016/j.jip.2023.107915

[17] Duan X-Z, Sun J-T, Wang L-T, Shu X-H, Guo Y, Keiichiro M, et al. Recent infection by Wolbachia alters microbial communities in wild Laodelphax striatellus populations. Microbiome. 2020;8:104. 32616041 10.1186/s40168-020-00878-x PMC7333401

[18] Thongsripong P, Chandler JA, Green AB, Kittayapong P, Wilcox BA, Kapan DD, et al. Mosquito vector-associated microbiota: metabarcoding bacteria and eukaryotic symbionts across habitat types in Thailand endemic for dengue and other arthropod‐borne diseases. Ecol Evol. 2018;8:1352–68. 29375803 10.1002/ece3.3676 PMC5773340

[19] Chandler JA, Liu RM, Bennett SN. RNA shotgun metagenomic sequencing of northern California (USA) mosquitoes uncovers viruses, bacteria, and fungi. Front Microbiol. 2015;6:185. 25852655 10.3389/fmicb.2015.00185 PMC4371751

[20] Hegde S, Khanipov K, Albayrak L, Golovko G, Pimenova M, Saldaña MA, et al. Microbiome Interaction Networks and Community structure from Laboratory-Reared and Field-Collected Aedes aegypti, Aedes albopictus, and Culex quinquefasciatus Mosquito vectors. Front Microbiol. 2018;9:2160. 30250462 10.3389/fmicb.2018.02160 PMC6140713

[21] Žiegytė R, Bernotienė R, Bukauskaitė D, Palinauskas V, Iezhova T, Valkiūnas G. Complete sporogony of *Plasmodium relictum* (lineages pSGS1 and pGRW11) in mosquito *Culex pipiens pipiens* form molestus, with implications to avian malaria epidemiology. J Parasitol déc. 2014;100(6):878–82. 10.1645/13-469.1 24979183

[22] Davis NM, Proctor DM, Holmes SP, Relman DA, Callahan BJ. Simple statistical identification and removal of contaminant sequences in marker-gene and metagenomics data. Microbiome. 2018;6:226. 30558668 10.1186/s40168-018-0605-2 PMC6298009

[23] Bolyen E, Rideout JR, Dillon MR, Bokulich NA, Abnet CC, Al-Ghalith GA, et al. Reproducible, interactive, scalable and extensible microbiome data science using QIIME 2. Nat Biotechnol août. 2019;37(8):852–7. 10.1038/s41587-019-0209-9 PMC7015180 31341288

[24] Callahan BJ, McMurdie PJ, Rosen MJ, Han AW, Johnson AJA, Holmes SP. DADA2: high-resolution sample inference from Illumina amplicon data. Nat Methods Juill. 2016;13(7):581–3. 10.1038/nmeth.3869 PMC4927377 27214047

[25] Katoh K, Misawa K, Kuma K, Miyata T. MAFFT: a novel method for rapid multiple sequence alignment based on fast Fourier transform. Nucleic Acids Res 15 Juill. 2002;30(14):3059–66. 10.1093/nar/gkf436 PMC135756 12136088

[26] Price MN, Dehal PS, Arkin AP. FastTree 2–approximately maximum-likelihood trees for large alignments. PLoS One 10 mars. 2010;5(3):e9490. 10.1371/journal.pone.0009490 PMC2835736 20224823

[27] Bokulich NA, Kaehler BD, Rideout JR, Dillon M, Bolyen E, Knight R, et al. Optimizing taxonomic classification of marker-gene amplicon sequences with QIIME 2’s q2-feature-classifier plugin. Microbiome 17 mai. 2018;6(1):90. 10.1186/s40168-018-0470-z PMC5956843 29773078

[28] Yarza P, Yilmaz P, Pruesse E, Glöckner FO, Ludwig W, Schleifer K-H, et al. Uniting the classification of cultured and uncultured bacteria and archaea using 16S rRNA gene sequences. Nat Rev Microbiol Sept. 2014;12(9):635–45. 10.1038/nrmicro3330 25118885

[29] Shannon CE. A Mathematical Theory of Communication. Bell Syst Tech J. 1948;27:379–423.

[30] Pielou EC. The measurement of diversity in different types of biological collections. J Theoretical Biology J Theoretical Biology. 1966;13:131–44.

[31] Faith DP. Conservation evaluation and phylogenetic diversity. Biol Conserv. 1992;61(1):1–10.

[32] DeSantis TZ, Hugenholtz P, Larsen N, Rojas M, Brodie EL, Keller K, et al. Greengenes, a chimera-checked 16S rRNA gene database and workbench compatible with ARB. Appl Environ Microbiol Juill. 2006;72(7):5069–72. 10.1128/AEM.03006-05 PMC1489311 16820507

[33] Bray JR, Curtis JT. An ordination of the upland forest communities of Southern Wisconsin. Ecol Monogr. 1957;27(4):325–49.

[34] Oksanen J, Blanchet FG, Friendly M, Kindt R, Legendre P, McGlinn D et al. vegan: Community Ecology Package. R package version 25 – 7 [Internet]. 2020; https://CRAN.R-project.org/package=vegan

[35] R Core Team. R: A language and environment for statistical computing. 2022;R Foundation for Statistical Computing, Vienna, Austria. https://www.R-project.org/

[36] Fernandes AD, Reid JN, Macklaim JM, McMurrough TA, Edgell DR, Gloor GB. Unifying the analysis of high-throughput sequencing datasets: characterizing RNA-seq, 16S rRNA gene sequencing and selective growth experiments by compositional data analysis. Microbiome. 2014;2:15. 24910773 10.1186/2049-2618-2-15 PMC4030730

[37] Aitchison J, Methodological B.). 1982.

[38] Friedman J, Alm EJ. Inferring correlation networks from genomic survey data. PLoS Comput Biol. 2012;8(9):e1002687. 23028285 10.1371/journal.pcbi.1002687 PMC3447976

[39] Bastian M, Heymann S, Jacomy M. Gephi: An open source software for exploring and manipulating networks. WebAtlas. 2009.

[40] Röttjers L, Vandeputte D, Raes J, Faust K. Null-model-based network comparison reveals core associations. ISME Commun 16 Juill. 2021;1(1):36. 10.1038/s43705-021-00036-w PMC9723671 37938641

[41] Anaconda Inc. Anaconda Software Distribution. Anaconda Documentation [Internet]. 2020; https://docs.anaconda.com/

[42] Peschel S, Müller CL, von Mutius E, Boulesteix A-L, Depner M. NetCoMi: network construction and comparison for microbiome data in R. Brief Bioinform 20 Juill. 2021;22(4):bbaa290. 10.1093/bib/bbaa290 PMC8293835 33264391

[43] Lhomme S. Analyse spatiale de la structure des reseaux techniques dans un contexte de risques. Cybergeo: European Journal of Geography. 2015.

[44] Csárdi G, Nepusz T, Csárdi. Gábor and Tamás Nepusz. The igraph software package for complex network research. (2006). InterJournal Complex Syst. 2006;1695(5):1–9.

[45] Mateos-Hernández L, Obregón D, Maye J, Borneres J, Versille N, de la Fuente J, et al. Anti-tick Microbiota Vaccine impacts Ixodes ricinus performance during feeding. Vaccines (Basel) 21 nov. 2020;8(4):702. 10.3390/vaccines8040702 PMC7711837 33233316

[46] Mateos-Hernández L, Obregón D, Wu-Chuang A, Maye J, Bornères J, Versillé N, et al. Anti-microbiota vaccines modulate the Tick Microbiome in a taxon-specific manner. Front Immunol. 2021;12:704621. 34322135 10.3389/fimmu.2021.704621 PMC8312226

[47] Minard G, Mavingui P, Moro CV. Diversity and function of bacterial microbiota in the mosquito holobiont. Parasit Vectors 20 mai. 2013;6:146. 10.1186/1756-3305-6-146 PMC3667145 23688194

[48] Douglas AE. The microbial dimension in insect nutritional ecology. Funct Ecol. 2009;23(1):38–47.

[49] Muturi EJ, Lagos-Kutz D, Dunlap C, Ramirez JL, Rooney AP, Hartman GL et al. Mosquito microbiota cluster by host sampling location. Parasit Vectors. 14 août. 2018;11(1):468. 10.1186/s13071-018-3036-9 PMC6092830 30107817

[50] Dickson LB, Jiolle D, Minard G, Moltini-Conclois I, Volant S, Ghozlane A, et al. Carryover effects of larval exposure to different environmental bacteria drive adult trait variation in a mosquito vector. Sci Adv août. 2017;3(8):e1700585. 10.1126/sciadv.1700585 PMC5559213 28835919

[51] da Silva H, Oliveira TMP, Sallum MAM. nov. Bacterial Community Diversity and Bacterial Interaction Network in Eight Mosquito Species. Genes (Basel). 2022 Nov 7;13(11):2052. 10.3390/genes13112052. PMID: 36360289; PMCID: PMC9690548. 10.3390/genes13112052 PMC9690548 36360289

[52] Banerjee S, Schlaeppi K, van der Heijden MGA. Keystone taxa as drivers of microbiome structure and functioning. Nat Rev Microbiol Sept. 2018;16(9):567–76. 10.1038/s41579-018-0024-1 29789680

[53] Tchioffo MT, Abate L, Boissière A, Nsango SE, Gimonneau G, Berry A, et al. An epidemiologically successful Escherichia coli sequence type modulates Plasmodium falciparum infection in the mosquito midgut. Infect Genet Evol Sept. 2016;43:22–30. 10.1016/j.meegid.2016.05.002 27154329

[54] Boissière A, Tchioffo MT, Bachar D, Abate L, Marie A, Nsango SE, et al. Midgut microbiota of the malaria mosquito vector Anopheles gambiae and interactions with Plasmodium falciparum infection. PLoS Pathog. 2012;8(5):e1002742. 22693451 10.1371/journal.ppat.1002742 PMC3364955

[55] Aranda-Díaz A, Willis L, Nguyen TH, Ho P-Y, Vila J, Thomsen T et al. Assembly of gut-derived bacterial communities follows « early-bird » resource utilization dynamics. bioRxiv. 14 janv 2023;2023.01.13.523996. 10.1016/j.cels.2025.101240 40157357

[56] Rasgon JL, Scott TW. Wolbachia and cytoplasmic incompatibility in the California Culex pipiens mosquito species complex: parameter estimates and infection dynamics in natural populations. Genet déc. 2003;165(4):2029–38. 10.1093/genetics/165.4.2029 PMC1462871 14704183

[57] Lee JM, Yek SH, Wilson RF, Rahman S. Characterization of the Aedes albopictus (Diptera: Culicidae) holobiome: bacterial composition across land use type and mosquito sex in Malaysia. Acta Trop. 2020;212:105683. 32888935 10.1016/j.actatropica.2020.105683

[58] Bian G, Xu Y, Lu P, Xie Y, Xi Z. The endosymbiotic bacterium Wolbachia induces resistance to dengue virus in Aedes aegypti. PLoS Pathog 1 avr. 2010;6(4):e1000833. 10.1371/journal.ppat.1000833 PMC2848556 20368968

[59] Coyte KZ, Rao C, Rakoff-Nahoum S, Foster KR. Ecological rules for the assembly of microbiome communities. PLoS Biol févr. 2021;19(2):e3001116. 10.1371/journal.pbio.3001116 PMC7946185 33606675

[60] Coyte KZ, Schluter J, Foster KR. The ecology of the microbiome: networks, competition, and stability. Sci 6 nov. 2015;350(6261):663–6. 10.1126/science.aad2602 26542567

