# Supplementary figures and images for: Nested patterns of commensals and endosymbionts in microbial communities of mosquito vectors
Source: BMC Microbiol. 2024 Oct 26;24:434. doi: 10.1186/s12866-024-03593-x (PMC11520040; doi:10.1186/s12866-024-03593-x)

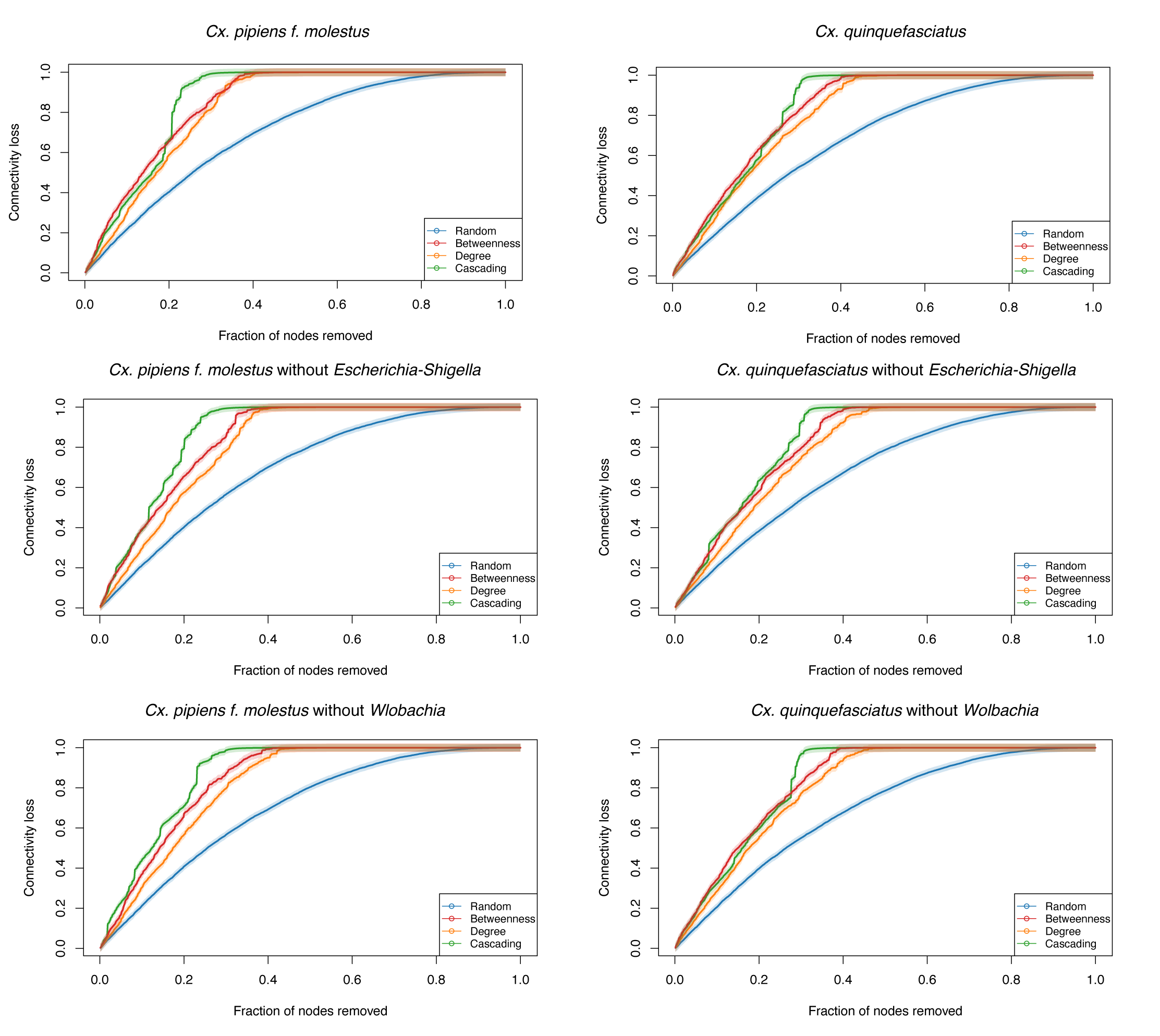

Supplement: Supplementary file 1 — Supplementary Material 1 [file 12866_2024_3593_MOESM1_ESM.png]

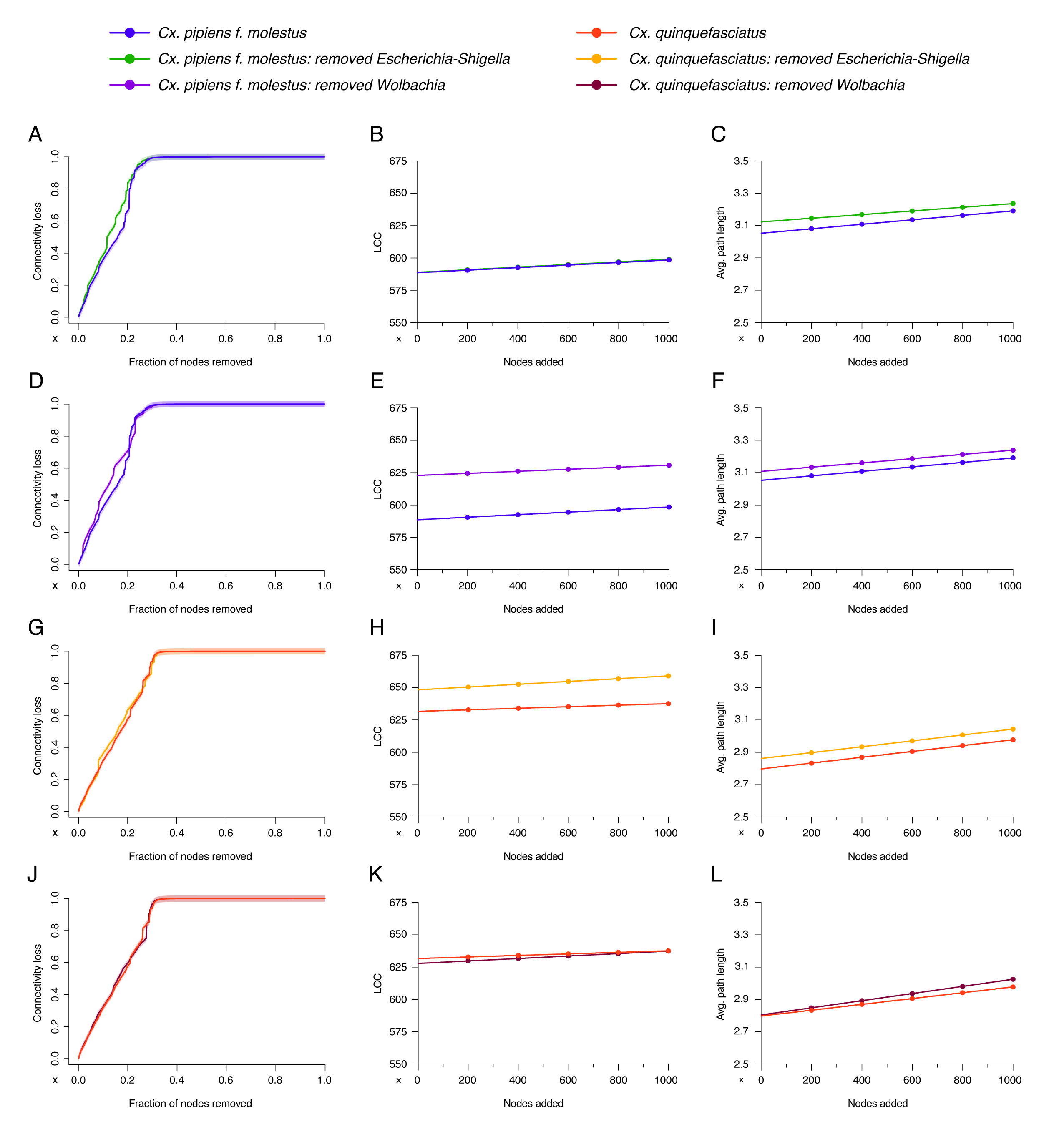

Supplement: Supplementary file 2 — Supplementary Material 2 [file 12866_2024_3593_MOESM2_ESM.png]

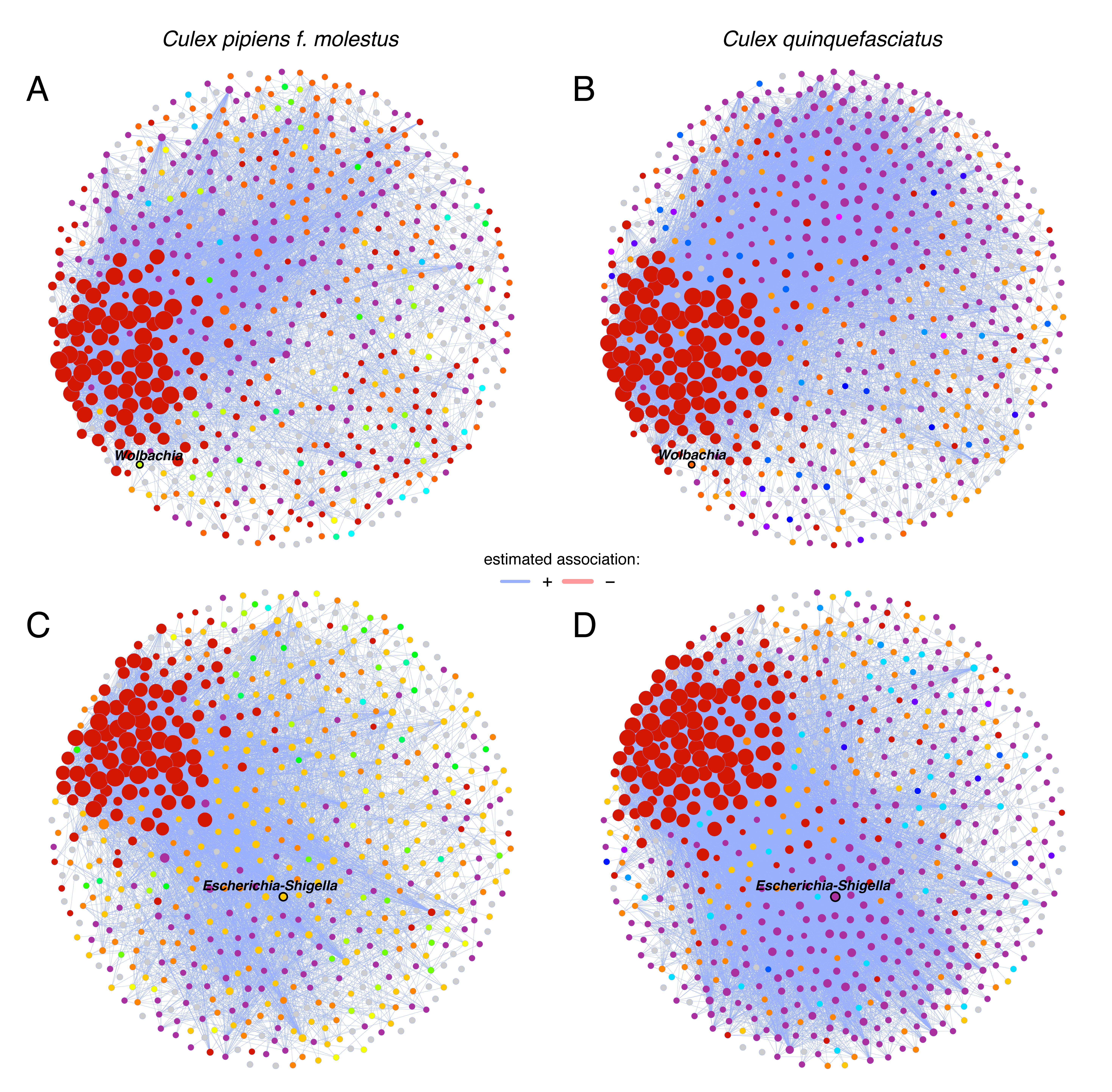

Supplement: Supplementary file 7 — Supplementary Material 7 [file 12866_2024_3593_MOESM7_ESM.png]
