# Supplementary material for: Nested patterns of commensals and endosymbionts in microbial communities of mosquito vectors
Source: BMC Microbiol. 2024 Oct 26;24:434. doi: 10.1186/s12866-024-03593-x (PMC11520040; doi:10.1186/s12866-024-03593-x)
Supplement: Supplementary file 3 — Supplementary Material 3 [file 12866_2024_3593_MOESM3_ESM.docx]

|  | ***Cx. pipiens* *molestus* with vs. without *Esherichia-Shigella*** | | | ***Cx. quinquefasciatus* with vs. without *Esherichia-Shigella*** | | | ***Cx. pipiens* *molestus* with vs. without *Wolbachia*** | | | ***Cx. quinquefasciatus* with vs. without *Wolbachia*** | | |
| --- | --- | --- | --- | --- | --- | --- | --- | --- | --- | --- | --- | --- |
| **Parameters** | **Jacc** | **P(<=Jacc)** | **P(>=Jacc)** | **Jacc** | **P(<=Jacc)** | **P(>=Jacc)** | **Jacc** | **P(<=Jacc)** | **P(>=Jacc)** | **Jacc** | **P(<=Jacc)** | **P(>=Jacc)** |
| Degree | 0.929 | 1 | 0*** | 0.907 | 1 | 0*** | 0.934 | 1 | 0*** | 0.916 | 1 | 0*** |
| Betweenness centr. | 0.694 | 1 | 0*** | 0.737 | 1 | 0*** | 0.715 | 1 | 0*** | 0.715 | 1 | 0*** |
| Closeness centr. | 0.943 | 1 | 0*** | 0.907 | 1 | 0*** | 0.962 | 1 | 0*** | 0.925 | 1 | 0*** |
| Eigenvec. centr. | 0.916 | 1 | 0*** | 0.925 | 1 | 0*** | 0.943 | 1 | 0*** | 0.934 | 1 | 0*** |
| Hub taxa | 0.894 | 1 | 0*** | 0.916 | 1 | 0*** | 0.885 | 1 | 0*** | 0.934 | 1 | 0*** |

Table S4. Jaccard indices for *Cx. pipiens* f. *molestus* and *Cx. quinquefasciatus* networks without *Escherichia-Shigella* vs. *Wolbachia.*
