# Supplementary material for: Nested patterns of commensals and endosymbionts in microbial communities of mosquito vectors
Source: BMC Microbiol. 2024 Oct 26;24:434. doi: 10.1186/s12866-024-03593-x (PMC11520040; doi:10.1186/s12866-024-03593-x)
Supplement: Supplementary file 4 — Supplementary Material 4 [file 12866_2024_3593_MOESM4_ESM.docx]

Table S3. Feature table of *Cx. pipiens f. molestus* and *Cx. quinquefasciatus* networks without *Escherichia-Shigella* vs. *Wolbachia*

| Network features | *Cx. pipiens f. molestus* | | *Cx. quinquefsciatus* | |
| --- | --- | --- | --- | --- |
|  | Removed *Esherichia-Shigella* | Removed *Wolbachia* | Removed *Esherichia-Shigella* | Removed *Wolbachia* |
| Nodes | 612 | 622 | 650 | 628 |
| Edges | 6579 | 6866 | 10438 | 10522 |
| Positive | 4253 (65%) | 4439 (65%) | 5789 (55%) | 5785 (55%) |
| Negative | 2326 (35%) | 2427 (35%) | 4649 (45%) | 4737 (45%) |
| Network diameter | 8 | 8 | 8 | 7 |
| Average degree | 21.5 | 22.077 | 32.117 | 33.51 |
| Weighted degree | 4.358 | 4.426 | 2.718 | 2.64 |
| Average path length | 3.138 | 3.147 | 2.875 | 2.819 |
| Modularity | 1.263 | 1.298 | 2.595 | 2.949 |
| Number of modules | 61 | 48 | 53 | 58 |
| Average clustering coefficient | 0.432 | 0.459 | 0.494 | 0.512 |
