# Supplementary material for: Nested patterns of commensals and endosymbionts in microbial communities of mosquito vectors
Source: BMC Microbiol. 2024 Oct 26;24:434. doi: 10.1186/s12866-024-03593-x (PMC11520040; doi:10.1186/s12866-024-03593-x)
Supplement: Supplementary file 6 — Supplementary Material 6 [file 12866_2024_3593_MOESM6_ESM.docx]

**Supplementary material legends**

**Figure S1**. The comparison of microbial arrangement between *Cx. pipiens* f*.* *molestus* (A, C) and *Cx. quinquefasciatus* (B, D) networks without *Escherichia-Shigella* (A, B) or *Wolbachia* (C, D). Bacterial taxa (family or genus level) with at least one connection are symbolized by nodes, whilst connected edges represent a significant correlation between them (SparCC, *p* < 0.05, weight ≥ 0.5 or ≤ -0.5). Node colours are based on determined clusters and sized according to the node’s eigenvector centrality. Positive (purple) or negative (red) correlations are shown by the colour of the edges.

**Figure S2.** Network tolerance to nodes removal. The robustness of the networks to directed, cascading, degree and random attacks was measured and compared in the natural networks, networks without *Escherichia-Shigella* or *Wolbachia.*

**Figure S3.** Network tolerance to nodes removal and addition in modified networks. (A, D, G, J) The resistance to cascading attack was measured in natural networks of *Cx. pipiens* f. *molestus* (A, D) and *Cx. quinquefasciatus* (G, J) and compared to their respective networks without *Escherichia-Shigella* (A, G) or *Wolbachia* (D, J). The robustness to nodes addition (from 0 to 1000) based on the size of the largest connected component (LCC) (B, E, H, K) and average path length (avg. path length) (C, F, I, L) was measured and compared between *Cx. pipiens* f. *molestus* or *Cx. quinquefasciatus* natural network to their respective networks without *Escherichia-Shigella* (B, C, H, I) and *Wolbachia* (E, F, K L). Different curve colours represent different groups.

**Table S1.** Bacterial taxa found as contaminants in the 16S rRNA gene sequencing datasets from mosquitoes of different species. Contaminants were statistically identified (TRUE) and removed from the 16S rRNA gene sequencing datasets using the decontam R package.

**Table S2.** List of shared and unique taxa present in the microbiota of *Cx. pipiens* f*. molestus* and/or *Cx. quinquefasciatus.*

**Table S3**. Feature table of *Cx. pipiens* f*. molestus* and *Cx. quinquefasciatus* networks without *Escherichia-Shigella* vs. *Wolbachia.*

**Table S4**. Jaccard indices for *Cx. pipiens* f. *molestus* and *Cx. quinquefasciatus* networks without *Escherichia-Shigella* vs. *Wolbachia.*
